# Supplementary material for: Immunoreactivity Analysis of MHC-I Epitopes Derived from the Nucleocapsid Protein of SARS-CoV-2 via Computation and Vaccination
Source: Vaccines (Basel). 2024 Oct 25;12(11):1214. doi: 10.3390/vaccines12111214 (PMC11598499; doi:10.3390/vaccines12111214)
Supplement: Supplementary file 1 [file vaccines-12-01214-s001.zip › vaccines-3245306-Supplementary Figures.pdf]

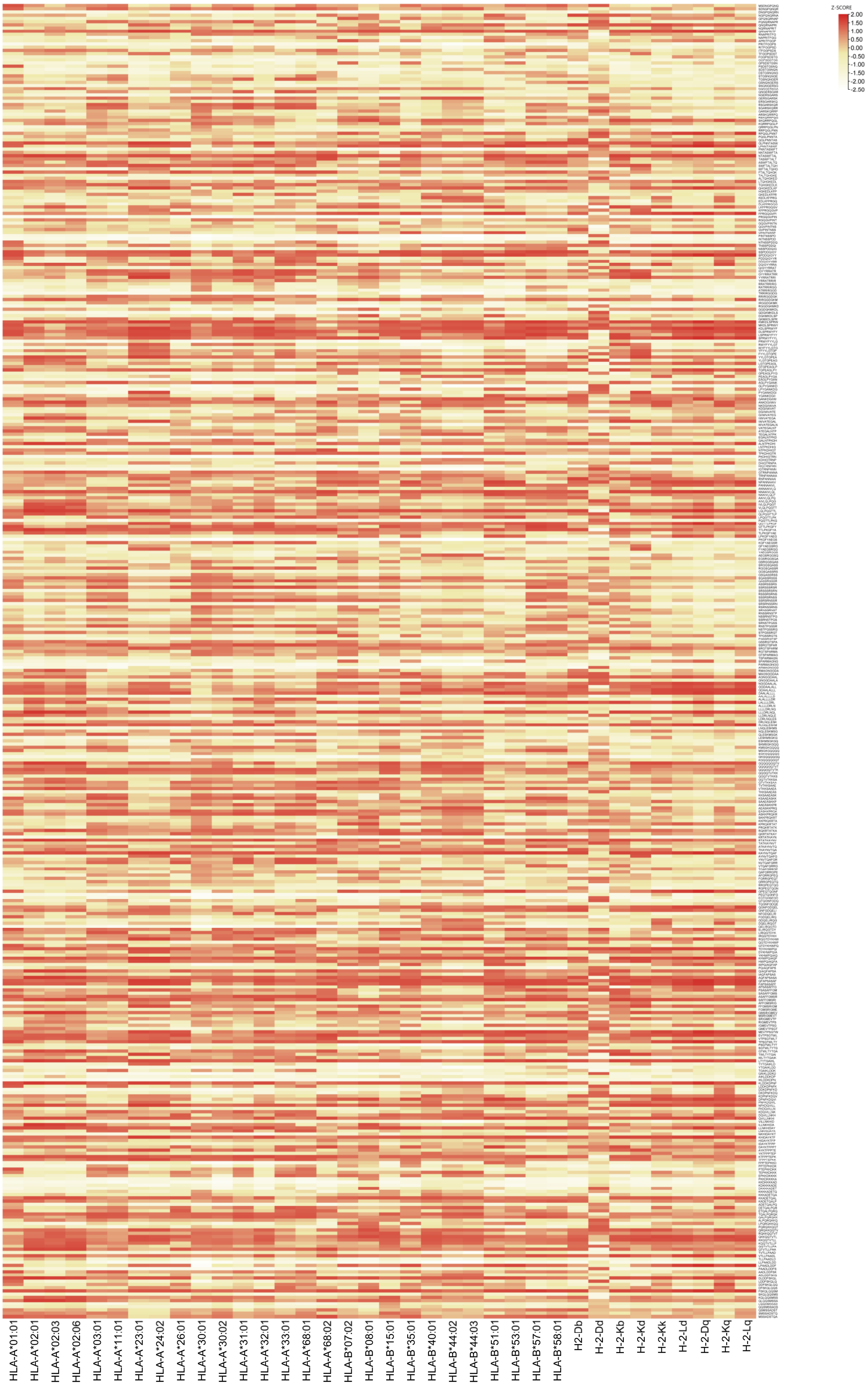

Supplementary Figure S1: Heatmap illustrating the interaction between MHC-I molecules and predicted epitopes. Columns and rows show MHC-I subtypes and epitopes, respectively. Darker colors represent a stronger interaction (lower %rank). The scale is plotted at the top right of the heat map, with values ranging from -2.5 to 2, and the color transitioning from white to yellow and finally to red. It represents

the affinity from low to high. Where white color represents MHC molecules binding to 9-mer peptide with low affinity and red color represents MHC molecules binding to 9-mer peptide with high affinity.

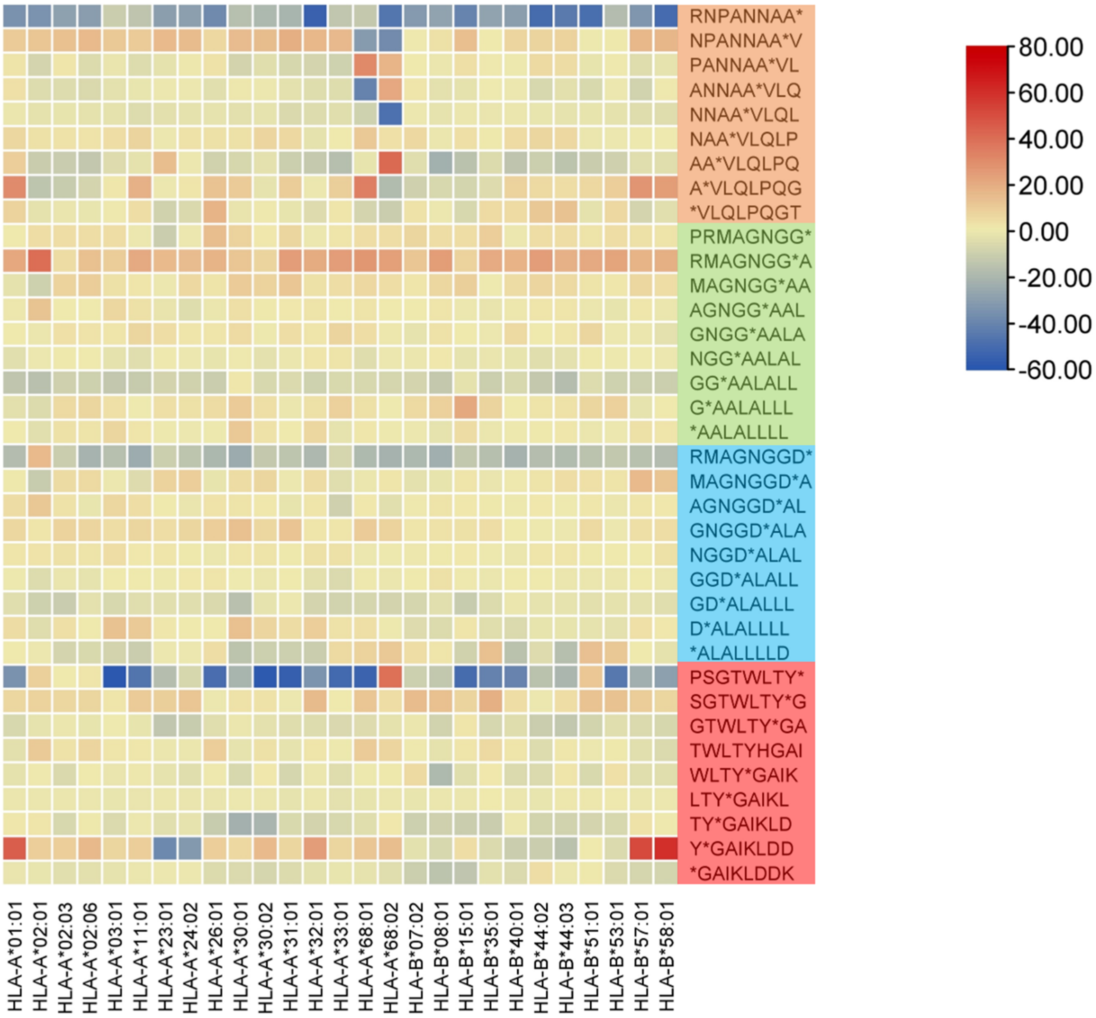

Supplementary Figure S2: The heatmap of 7 consecutive peptide sequences containing each mutation site. TBtools drew the heatmap of the binding affinity delta value between 76-118 and variants of corresponding HLA-I and 9-mer epitopes, where the binding affinity took the logarithm of base 2. The delta value of %Rank as minus represented the epitope of strain 76-118 with better affinity. And contrarily, plus values stood for that of variants acquiring better binding performance with corresponding MHC-I molecule.
